# Observations on Antisoric Preparations

**Published:** 1802-08-01

**Authors:** John Ring

**Affiliations:** Surgeon


### *Observations on Antisoric Preparations; communicated by Mr.* JOHN RING, *Surgeon.*

WHEN I communicated a remedy for a loathſome diſeaſe, which has ſtood the teſt of twenty-five years experience, and not failed in a ſingle inſtance, I added a caution concerning its uſe, to prevent the only inconvenience with which it is attended. This caution, if obſerved, renders it as ſafe an application as any other of equal efficacy in the whole circle of medicine.

An anonymous Author of a Letter in the laſt Number of your Journal, has expreſſed a diſlike to the formula, becauſe he has ſeen unpleaſant effects produced by the external application of hydrargyrus muriatus in cutaneous diſeaſes. Who has not ſeen unpleaſant effects from the uſe of every powerful medicine, whether employed externally or internally? If the abuſe of a remedy is to be conſidered as an argument againſt its uſe, bark, opium, and murcury muſt be baniſhed from the Materia Medica; and any man who gives antimonial powder in a fever, or antimonial wine as an emetic, ought to be condemned to the gallies; as he would have been two hundred and fifty years ago, had he dared to commit that crime at Rome.

Lotions, in which hydrargyrus muriatus is an ingredient, are very apt to inflame and excoriate any tender part; and it was this circumſtance that firſt ſuggeſted to me the idea of uſing it in an unctuous form.

Your Correſpondent ſtrains at a gnat and ſwallows a camel. He objects to the external uſe of ſublimate, becauſe he has ſeen it, when incautiouſly applied, produce inflammation of the ſkin, and excoriation; yet recommends calomel to be exhibited internally; a medicine which, however valuable in ſome caſes, is in cutaneous complaints unneceſſary; a medicine which has ſent many a victim to an untimely grave.

Your Correſpondent has been more fortunate than I ever was, in commonly curing the itch by two or three applications of white precipitate. He has alſo been more fortunate in correcting the unpleaſant and diſgraceful ſmell of even that preparation of ſulphur which he recommends. It is, however, only juſtice to the inexperienced practitioner, to put in a caveat againſt his placing two much confidence in the reſult of your Correſpondent's experience. Had not the mild preparations, ſo highly extolled by your Correſpondent, frequently failed in my practice and that of others, even when perſiſted in for a much longer period, I ſhould never have thought of employing what was likely to be attended with the leaſt hazard, even to the moſt ſuperficial part of the human frame.

Your polite attention to my former requeſt, and your ready inſertion of the memoir which I tranſmitted, claim the performance of the promiſe I then made.

The cure of gonorrhœa is rendered much more ſafe, pleaſant and expeditious, by the uſe of injections; which are now univerſally employed by the moſt expert practitioners. Neverthelefs, the beſt formula for that complaint in general, is, with regard to the majority of medical men, ſtill a deſideratum.

The following was communicated to me ſeveral years ago, by a gentleman not of the profeſſion; who informed me, that having been peculiarly unfortunate, and often under the neceſſity of having recourſe to remedies of this kind, he found it much more efficacious than any other which he had employed.

℞. Calomel. pul. gum. Arab. a. ʒij. Aquæ ℥vijſs. M.

It would be wrong to deny that this compoſition ſometimes proves too ſtimulating; an objection to which every remedy is liable in proportion to its efficacy. No man, however, ought to practiſe phyſic or ſurgery, who is not a judge of the caſes in which the injection ought to be uſed in a milder form; and of the means neceſſary to ſubdue the ſymptoms, which ariſe from its improper uſe.

Calomel, exhibited internally in this complaint, has been the death of thouſands. I hope, in this enlightened age, we ſhall ſee that practice quite exploded. To this end, ſo devoutly to be wiſhed, as well as to a general reform in the practice of phyſic, an Herculean labour, for which the united talents of all the members of the medical profeſſion are required, no Publication has contributed more, or is likely to contribute more, than your own.

If any internal medicine is adminiſtered, perhaps no one is more proper than the following.

℞. Nitri purif. ʒj. Pul. gum. Arab. ſacchari a. ʒſs. Hydrarg. ſulph. rubri gr. vj. M. Divide in pul. vj. quorum capiat j in aqua omni mane.

——— Si quid noviſti rectius iſtis,

Candidus imperti; ſi non, his utere mecum.

*New Street, Hanover Square,*

*June 17, 1802.*

*A Case*

